# Automated Workflow for Somatic and Germline Next Generation Sequencing Analysis in Routine Clinical Cancer Diagnostics

**DOI:** 10.3390/cancers11111691

**Published:** 2019-10-30

**Authors:** Lucia Anna Muscarella, Federico Pio Fabrizio, Maria De Bonis, Maria Teresa Mancini, Teresa Balsamo, Paolo Graziano, Flavia Centra, Angelo Sparaneo, Domenico Trombetta, Antonio Bonfitto, Vito Scagliusi, Pietro Larizza, Ettore Domenico Capoluongo, Vito Michele Fazio

**Affiliations:** 1 Laboratory of Oncology, Fondazione IRCCS Casa Sollievo della Sofferenza, 71013 San Giovanni Rotondo (FG), Italy; fp.fabrizio@operapadrepio.it (F.P.F.); t.balsamo@operapadrepio.it (T.B.); f.centra@operapadrepio.it (F.C.); a.sparaneo@operapadrepio.it (A.S.); d.trombetta@operapadrepio.it (D.T.); 2 Department of Laboratory Medicine of the ‘Agostino Gemelli’ Foundation in Rome, 00168 Rome, Italy; mari.debonis86@gmail.com; 3 MASMEC S.p.A, 70026 Modugno (BA), Italy; mariateresa.mancini@masmec.com (M.T.M.); vito.scagliusi@masmec.com (V.S.); piero.larizza@masmec.com (P.L.); 4 Unit of Pathology, Fondazione IRCCS Casa Sollievo della Sofferenza, 71013 San Giovanni Rotondo (FG), Italy; p.graziano@operapadrepio.it (P.G.); antonio.bonfitto@operapadrepio.it (A.B.); 5 Department of Molecular Medicine and Medical Biotechnologies, University Federico II-CEINGE, 80145 Naples, Italy; capoluongo@ceinge.unina.it; 6 Department of Medicine, R.U. in Molecular Medicine and Biotechnology, University Campus Bio-Medico of Rome, 00128 Rome, Italy

**Keywords:** FFPE, DNA extraction, automation, library preparation, NGS

## Abstract

Thanks to personalized medicine trends and collaborations between industry, clinical research groups and regulatory agencies, next generation sequencing (NGS) is turning into a common practice faster than one could have originally expected. When considering clinical applications of NGS in oncology, a rapid workflow for DNA extraction from formalin-fixed paraffin-embedded (FFPE) tissue samples, as well as producing high quality library preparation, can be real challenges. Here we consider these targets and how applying effective automation technology to NGS workflows may help improve yield, timing and quality-control. We firstly evaluated DNA recovery from archived FFPE blocks from three different manual extraction methods and two automated extraction workstations. The workflow was then implemented to somatic (lung/colon panel) and germline (*BRCA1/2*) library preparation for NGS analysis exploiting two automated workstations. All commercial kits gave good results in terms of DNA yield and quality. On the other hand, the automated workstation workflow has been proven to be a valid automatic extraction system to obtain high quality DNA suitable for NGS analysis (lung/colon Ampli-seq panel). Moreover, it can be efficiently integrated with an open liquid handling platform to provide high-quality libraries from germline DNA with more reproducibility and high coverage for targeted sequences in less time (*BRCA1/2*). The introduction of automation in routine workflow leads to an improvement of NGS standardization and increased scale up of sample preparations, reducing labor and timing, with optimization of reagents and management.

## 1. Introduction

Next generation sequencing (NGS) based technologies have been revolutionizing knowledge in cancer genomics and now represent valuable tools to characterize the molecular landscape of cancer genomes in different tumor types [1]. Due to its high throughput, NGS has also been rapidly adopted in clinical oncology practice to perform simultaneous analyses at both germline and somatic levels on several genes or gene regions, by means of a single test starting from very little input material [2]. Its use to identify germline (inherited) *BRCA1/2* mutations is currently a gold standard and different panels are used in oncology for predicting somatic (acquired) druggable and resistance mutations in some cancer settings. Formalin-fixed paraffin-embedded (FFPE) human tissues actually represent the most used source of genetic and epigenetic data in oncology by NGS analysis for diagnostic and translational research purposes [3]. In this context, quality control of the starting materials effects downstream workflows and impacts the value of NGS data. The sensitivity and specificity of these new high-throughput assays and the limited amount of biological materials give a continuous challenge regarding the choice of proper extraction method to rapidly obtain the most satisfactory yield and purity of DNA. The quality of DNA extracted from FFPE tissues is in fact variable and depends on many factors, such as the time and procedure of tissue fixation, embedding, and the long-term storage methods [4,5]. Despite good preservation of histological and cytological features of fixation, this procedure in fact frequently generates DNA impurities and fragmentation due to DNA induced cross-links between proteins and DNA and scission at the phosphodiester backbone of DNA [6]. The most common low-cost and “home-made” procedure for DNA recovery used in many laboratories is still phenol/chloroform extraction [7]. Alternatively, there are a lot of ready-to-use commercially available kits dedicated to DNA extraction from FFPE tissues. They can be divided into two subtypes based on silica exchange resins or magnetic bead-based technologies. To improve the success rate of molecular analysis, many procedures and commercial kits still require a great amount of initial specimen to be processed to compensate for the low yields of DNA [8,9].

In addition to the high quality of nucleic acid, NGS requires the conversion of the source nucleic acid material into standard libraries suitable for loading onto a sequencing instrument. Therefore, library preparation can result as the main bottleneck in the NGS workflow and represents one of the most critical, hands-on, and time consuming steps. Each library must be prepared in an independent well, increasing the number of hours required for a sequencing run and the risk of human-introduced error. To optimize the process and reduce preparation time, one rational option is automated library preparation [10], which offers significant advantages over the manual preparation of samples. Through automation, human error can be reduced and experimental costs can consequently be lowered. Additionally, automation may significantly eliminate the variability found in manual processing, providing identical conditions to create more reproducible sample processing [11]. 

In the present work, we aim to optimize a rapid and efficient workflow to combine automatic DNA extraction and library preparation by using two different training sets of FFPE tissues and peripheral blood samples from patients with tumors. Opting for an automated setup will allow us to address the problems associated with manual protocols (i.e., risk of cross-contamination, manual mix-up, scaling up, errors in aliquoting procedures, timing), helping to achieve consistent data, minimize errors, and increase speed and throughput.

We compared and evaluated the performance of two automated DNA extraction systems, King Fisher Duo and OMNIA Prima, and three of the most commonly used manual DNA extraction systems: phenol/chloroform (PC), GeneRead DNA FFPE Kit (GR) and MagMAX FFPE Total Nucleic Acid Isolation Kit (MM). Specifically, three different methods to assess DNA quantity (NanoDrop/ND-1000 and Qubit 3.0) and quality (Genomic DNA ScreenTape system and GeneRead DNA QuantiMIZE Assay, Qubit 3.0) were used to check the DNA extracted from FFPE tissue blocks and find the most suitable and reliable pre-analytical workflow for NGS analysis. In addition, we described a rapid and automated method to prepare a Multiplicom NGS-based protocol for the Illumina MiSeq platform (Illumina, San Diego, CA, USA) for *BRCA1/2* genes using the OMNIA MASMEC System (Modugno, Bari, Italy), which is routinely used in our molecular diagnostic lab, and compared it to manual sample preparation.

## 2. Results

### 2.1. Manual vs. Automatic DNA from FFPE Tissue Extraction

A total of six FFPE tissue slides were extracted using each of the manual and automatic methodologies for the evaluation of DNA recovery and the results were compared. Each pair of tissues (tumor/paired normal tissue) were derived from the same patient with different times of formalin fixation (24 h, 48 h, 72 h). The first slide and the last slide were taken from each specimen and subsequently examined by staining with Hematoxylin&Eosin to verify the presence of cellular material in all sections. DNA extracts from the same samples were qualitatively and quantitatively evaluated (Table 1, Table 2, Table 3 and Table 4). All samples from the manual and automatic procedures were firstly loaded on a TapeStation 2200 instrument to visualize the amount of degradation by estimating the genomic DNA size range and DNA integrity number (DIN). 

**Figure 1:**
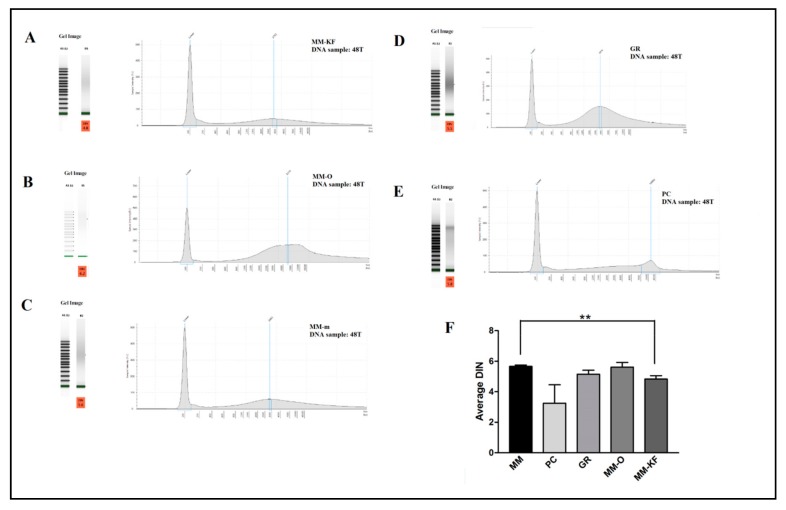
Five representative DNA samples from the same tissue block visualized using the TapeStation instrument. Profile of DNA extracted from the 48T sample using: (**A**) MM-KF, (**B**) MM-O, and (**C**) MM manual. Profile of DNA extracted from the 48T sample using: (**D**) GR kit, and (**E**) PC procedures. (**F**) Comparative results of DIN values (mean ± SE) for all six DNA samples from FFPE samples (24N, 24T, 48N, 48T, 72N, 72T) obtained by different extraction methods. ** *p* < 0.001, *t*-test. SE, standard error.

Figure 1 shows the micro-electrophoresis runs results of the DNA from tumor after 48 h of formalin inclusion (48T). All samples showed a good general DNA quality with a DIN value >4.4. No significant differences were observed, except for the DNA extracted by the PC procedure which failed to generate a DIN value in 2/6 samples and for a third sample generated a value of 1.9 (Table 4). We can conclude that any method worked about as well as the other methods.

**Figure 2:**
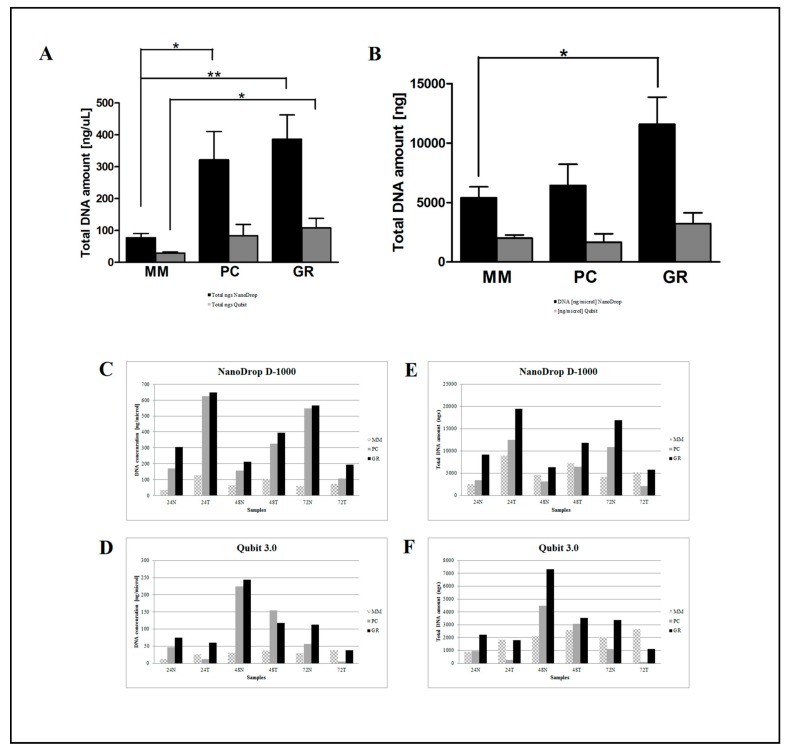
Comparison of the three manual DNA extraction systems (MM, PC and GR). Mean of DNA concentration (ng/μL) and total DNA (ng) as measured by the NanoDrop D-1000 spectrophotometer (**A**) and Qubit 3.0 fluorometer (**B**)**.** Total DNA amount (ng) measured by the NanoDrop D-1000 spectrophotometer (**E**) and Qubit 3.0 fluorometer (**F**) and DNA concentration (ng/μL) measured by the NanoDrop D-1000 spectrophotometer (**C**) and Qubit 3.0 fluorometer (**D**) for the 24N, 24T, 48N, 48T, 72N, and 72T samples. * *p* < 0.05, ** *p* < 0.001, *** *p* < 0.0001 *t*-test.

**Figure 3:**
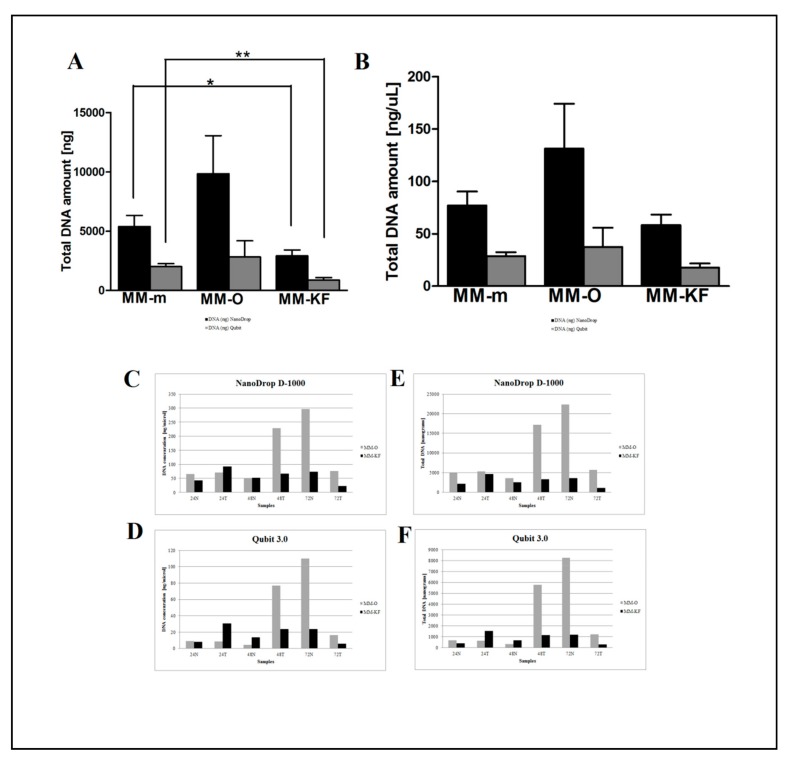
Comparison of two automatic DNA extraction systems (OMNIA Prima and King Fisher Duo). DNA concentration (ng/μL) measured by the NanoDrop D-1000 spectrophotometer (**A**) and Qubit 3.0 fluorometer (**B**). DNA (ng/μL) measured by the NanoDrop D-1000 spectrophotometer (**C**) and Qubit 3.0 fluorometer (**D**) for the 24N, 24T, 48N, 48T, 72N, and 72T samples. Total DNA amount (ng) measured by the NanoDrop D-1000 spectrophotometer (**E**) and Qubit 3.0 fluorometer (**F**) for the 24N, 24T, 48N, 48T, 72N, and 72T samples. * *p* < 0.05, ** *p* < 0.001, *** *p* < 0.0001 *t*-test.

DNA samples were then quantified by the NanoDrop D-1000 spectrophotometer and Qubit 3.0 fluorometer to estimate the absolute DNA concentration. The quantity and quality of extracted DNA samples by both manual and automatic procedures were compared across all samples. Less degradation and higher DNA molecular weight can be observed between DNA samples extracted using the three manual and automatic procedures (Figure 2 and Figure 3).

**Figure 4:**
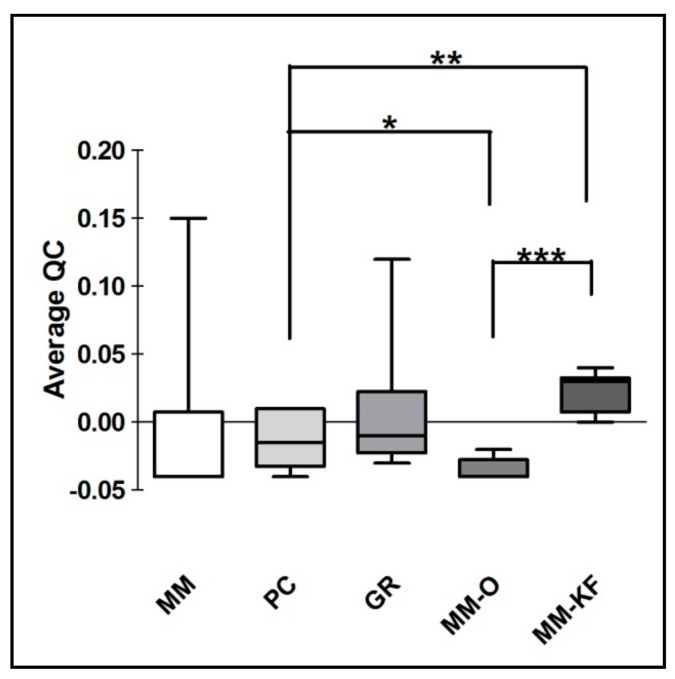
QuantiMIZE QC values comparison for DNA extracted using both manual procedures (MM, PC and GR) and automatic workstations (MM-O and MM-KF). * *p* < 0.05, ** *p* < 0.001, *** *p* < 0.0001 *t*-test.

However, there was a significant difference in the DNA yield per section between manual methods, both in terms of total nanograms and DNA concentration (ng/μL) (Figure 2). As expected, the number of samples measured with the fluorometric assay was generally lower than those with the spectrophotometric assay due to DNA fragmentation affecting the DNA extracted from FFPE samples. DNA degradation, in fact, does not affect the concentration measured with the NanoDrop spectrophotometer and the DNA concentration might even be overestimated due to the presence of single-stranded DNA in the solution [12]. Looking at each samples, both the total amount and the concentration of DNA extracted by GR were the highest in all samples regardless of tumor size, whereas the purity ratio at 260/280 nm ranged from 1.7 to 2.0 for all six samples and for all the methods employed. Therefore, all samples obtained by the three different investigated procedures would be typically considered as pure. The lowest A260/280 ratios obtained for DNA samples extracted by MM (1.76 ± 0.02) indicates a higher protein contamination level, probably resulting from the inefficient purification step. A variety of incubation times has been suggested for the deparaffinization and proteinase K digestion since a longer time should impact the retrieved DNA [13,14]. The GR and PC procedures recommended a deparaffination step resulting in significantly higher amounts of extracted DNA from the same amount of starting material. By contrast and for the same reason, DNA extracted by MM generally resulted in lower fragmentation than GR and PC (less difference between NanoDrop and Qubit dosages). As shown in Figure 3, focusing on the two automatic procedures, the amount of DNA extracted by the OMNIA Prima was equal or higher in all samples and no differences were observed when compared to the manual MM procedure. By contrast, KF extracts showed a significantly lower recovery of total DNA amount (Table 2 and Table 3). No differences regarding the fixation time (24 h, 48 h or 72 h) were observed, thus confirming that a variable time of 24–72 h used in the routine procedure for tissue handling does not significantly affect the quality and quantity of extracted DNA [15,16,17]. Finally, to measure the performance of DNA in downstream PCR, each sample across all extraction methods was amplified by real-time PCR with the QuantiMIZE assay according to manufacturer’s instructions (Table 4). Quality control (QC) values for all samples determined by QuantiMIZE assay in a real-time PCR setting showed acceptable QC values of <0.04 (Figure 4). Since the ability of the DNA to be amplified by PCR depends on its purity and absence of common contaminants (such as xylene, alcohols and salts), we can conclude that all methods used provided good quality DNA suitable for downstream PCR-based methodologies.

**Figure 5:**
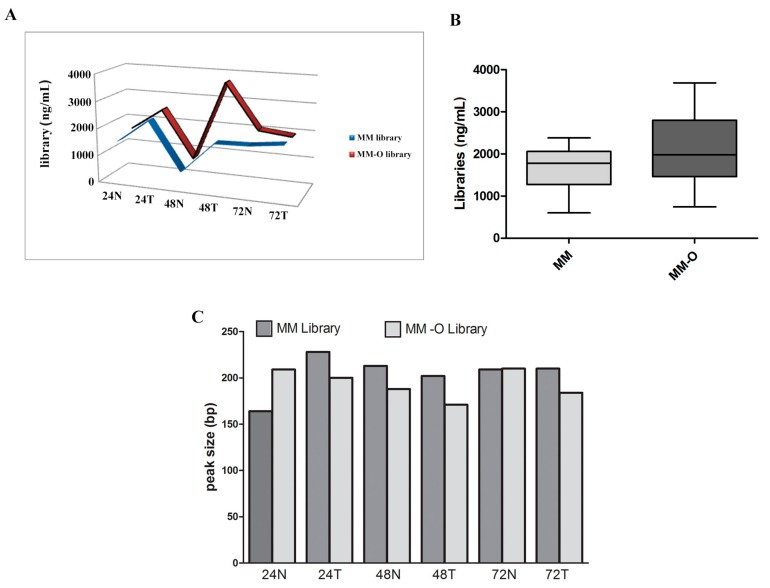
Library concentrations and distributions relative to the Colon and Lung Panel assay. Panels (**A**) and (**B**) show the concentrations of libraries obtained from samples manually (MM) and automatically (MM-O). Panel (**C**) represents the patterns of peak size of the same prepared samples (MM and MM-O).

The qualitative evaluation of NGS libraries from manual (MM) vs. automatic (MM-O) DNA FFPE tissue extraction methods was performed by the TapeStation System (Agilent Technologies) and a Qubit^®^ 3.0 Fluorometer. Similar quality performance in terms of amplicons pattern and library concentrations was observed. The means and standard deviation of library concentrations was 1665 ± 243.7 ng/µL and 2098 ± 396.6 ng/µL for manual and automated preparations, respectively, with no significant difference. The libraries obtained with the automated method were characterized by a similar uniform distribution of concentrations compared to the manual preparation (Figure 5). Finally, technical manual preparation on the bench required about two working days, while the automated procedure took no more than one day.

### 2.2. NGS Automatic Performance Workflow

**Figure 6:**
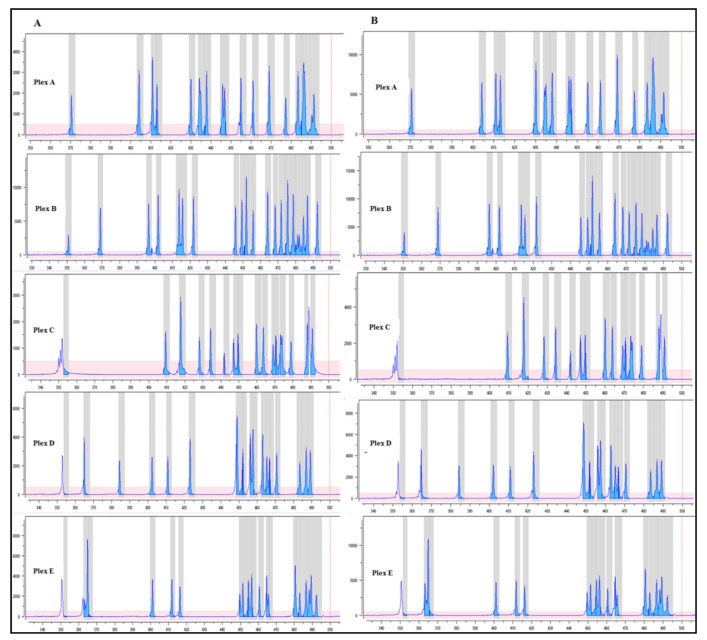
Fragment analysis profile. Quality of the multiplex PCR based on the pattern of amplicons of a five plex relative to the *BRCA1/2* MASTR Dx assay (Multiplicom). Panel (**A**) shows the profile for each plex of a sample manually prepared. Panel (**B**) represents the pattern of the same sample prepared by the automated method. On the x-axis, base pairs; y-axis, fluorescence intensity (RFU).

The qualitative evaluation of NGS libraries from peripheral blood DNA was performed by fragment analysis, as reported in Figure 6. Both manual and automated library preparation methods showed similar quality performances in terms of the pattern of the amplicons. Of note, the peak fluorescence intensities associated with automated library preparation were higher than those prepared manually.

**Figure 7:**
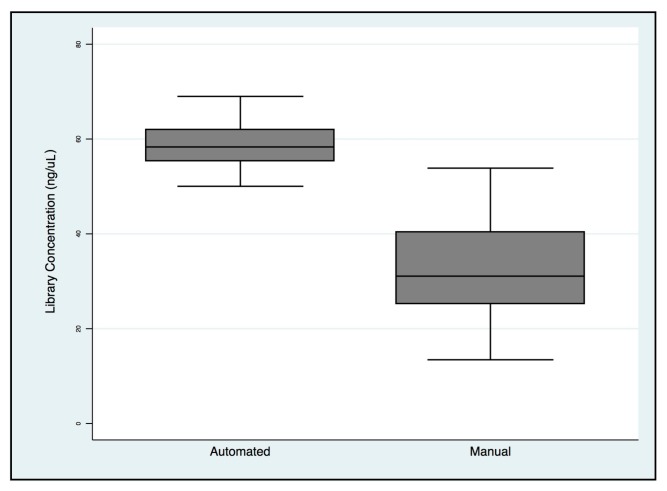
*BRCA1/2* libraries concentration comparison between manual and automated preparation workflows.

The means and standard deviations of library concentrations were 32.8 ± 10.9 ng/μL and 58.9 ± 4.9 ng/μL for manual and automated preparations, respectively, with a statistically significant difference (Figure 7).

**Figure 8:**
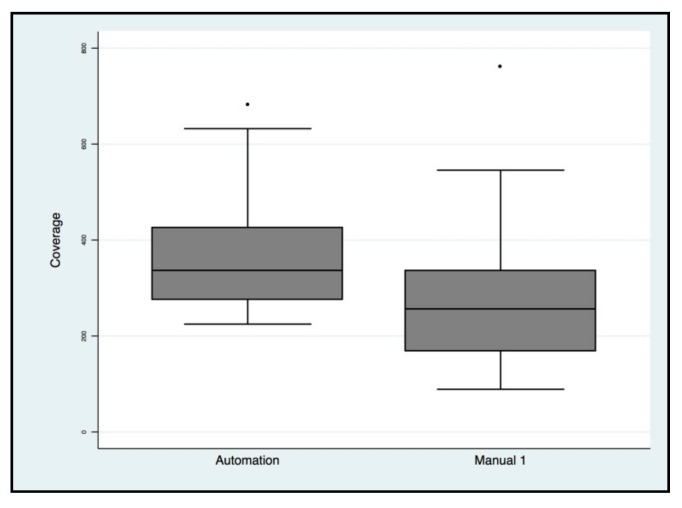
Evaluation of the uniformity of *BRCA1/2* library concentration by PicoGreen^®^ dsDNA quantitation assay.

As depicted in Figure 8, the libraries obtained with the automated method are characterized by a more uniform distribution of concentrations compared to manual preparation.

**Figure 9:**
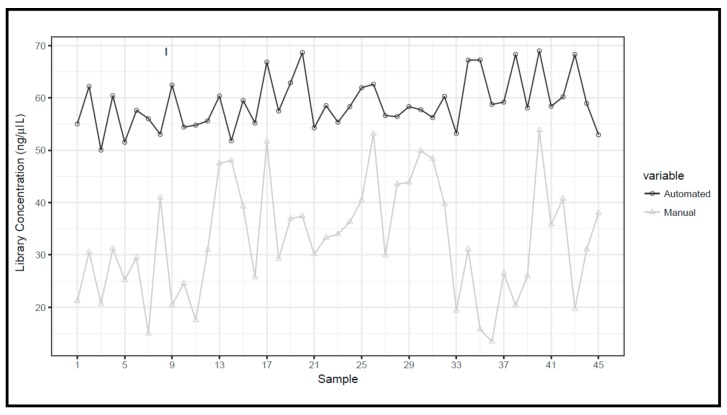
Next generation sequencing (NGS) coverage comparison of manual protocols vs. automated protocols for the *BRCA1/2* libraries.

Targeted NGS analysis was performed for all samples and the read coverage parameter of two types of libraries was compared. Particularly, the means and standard deviations were 269× ± 136 for manual protocols and 365× ± 112.8 for automated protocols, with a statistically significant difference (*p* = 0.0002) (Figure 9). No evidence of cross-contamination emerged by bioinformatic analysis (BAM, Binary Alignment Map files were evaluated by an expert bioinformatician), not only for the biological samples sequenced but also for the blank samples which were used to verify possible mix-ups or contamination events for both methods.

Finally, an overview of the output data from the NGS run is also reported in Table 5 in order to show the quality of the NGS parameters such as cluster passing filter, QC30 and error rates. The differences, although not significant between manual and automated library preparation, are reported. Particularly, the percentage of read passing filters were 92% and 85.9% for the libraries obtained with the automated protocol and the manual protocol, respectively. The report also demonstrated a Q30 score distribution of 85% for automated preparation and 80% for manual preparation. A total of 4 Gbyte was generated for this run with an error rate less of than 0.15% and 0.18% for automated and manual preparation, respectively. These values, in general, showed that the automated protocol performs better than the manual, although without statistically different results. 

## 3. Discussion

Sample acquisition and preparation is the most time-consuming step for molecular analyses of solid tumors. In this context, formalin fixation and paraffin embedding represent the most reliable ways to preserve tissues for a long time since FFPE samples are stable for decades [18]. However, the harmonization of this step is still challenging. Tissue extraction presents a distinct set of problems not applicable to blood or body fluids: formalin treatment cross-links biological molecules such as DNA and proteins [19] and induces consistent fragmentation of both DNA and RNA, leading to poor performance in downstream analyses [20,21]. Based on this assumption, it is obvious that it is necessary for the implementation of DNA analysis in the laboratory, to carefully evaluate the analytical platform, bioinformatics pipeline and standardization of the pre-analytical steps and tools for DNA extraction of FFPE samples. 

In the present study, we tested the performance of the most commonly used commercial kits and protocols (GR, MM, and PC) working on validated automatic pipelines (OMNIA Prima and King Fisher Duo). Serial extractions of DNA from a training set of paired tumor/normal tissues fixed in formalin for different times were performed. Comparison of the four quantification and qualification systems showed inter-method variation but all systems provided detailed information about the extracted DNA. The concentration of double stranded DNA measured by the fluorometric Qubit method seems to be the most useful measurement of amplifiable DNA; therefore, it can be integrated with real-time PCR amplification in the QuantiMIZE assay. The differences between Qubit and Nanodrop measurements may be explained by the fact that the Nanodrop instrument measures both single and double stranded DNA [21,22]. We observed that all extraction methods performed well, generating extracted DNA of high molecular weight with no heavy fragmentation; this feature is essential in whole genome sequencing approaches. GR was the most rapid manual method that was able to provide good DNA in terms of quality and quantity on all six samples, regardless of the conventional fixation times of tissues. Regarding the automatic procedures, OMNIA Prima showed higher quality results in DNA recovery. This automation solution enables a cost-effective preparation of samples with minimal hands-on time with an extraction yield and purity similar to those obtained by manual extraction using the same chemistry. Moreover, it is controlled by user-friendly software, with intuitive graphics for remote control and a simple interface that allows us to program and to personalize all of the workstation’s activities. This study also shows that the optimized pre-analytical step can be suitable for NGS library preparation using the AmpliSeq chemistry for massively parallel sequencing on Ion Torrent NGS platforms. The yield obtained using DNA extracted with the automatic protocol did not significantly differ from those obtained using DNA extracted with the manual protocol.

In the NGS workflow, the library preparation is one of the most time-consuming and laborious steps. Here, we describe a new automated solution for fast and reproducible *BRCA1/2* library preparation for NGS using a robotic workstation. In particular, we compared the reproducibility, reliability and quality of the DNA libraries and sequencing data produced using the automated protocol compared to the manual protocol. The introduction of these machines in our routine workflow led to an improvement in NGS standardization. Thanks to the perfect ratio of magnetic beads and the amount of DNA, libraries obtained with the MASMEC system showed a higher quantity and quality compared to the manual protocols. Consequently, our findings resulted in a higher quality of sequencing data. Automated sample preparation has significant advantages over manual preparation and the use of an automated workstation can be easily scaled to prepare up to 12 samples simultaneously. Furthermore, through automation, we achieved consistent optimization of reagents and management of laboratory professionals.

## 4. Materials and Methods 

### 4.1. DNA Extraction Workflow

#### 4.1.1. Biological Sample Collection and Macroscopic Evaluation

A set of 3 randomly anonymized tumors and matched normal tissues distant from tumors of the human gastrointestinal tract were collected according to the guidelines of the Local Ethical Committee at the Unit of Pathology of Istituto di Ricovero e Cura a Carattere Scientifico (IRCCS) Casa Sollievo della Sofferenza Hospital, Italy. Tissues were fixed after excision in 10% neutral buffered formalin at three different time points (24 h, 48 h and 72 h) before machine processing and embedding into paraffin. 

Ten micrometer thick sections were macro-dissected from paraffin blocks using a microtome with disposable blades. The first and last slides were stained with hematoxylin and eosin (H&E) in order to verify the absence of a significant portion of necrosis that could affect DNA recovery. DNA was extracted from the 10 μm FFPE sections. 

An additional training set of peripheral blood samples from 45 ovarian cancer patients that were referred to the Department of Clinical Molecular and Personalized Diagnostics of the Hospital ‘Agostino Gemelli’ Foundation in Rome, Italy, were collected after signing the appropriate informed consent. The study was conducted in accordance with the Declaration of Helsinki, and the protocol (Protocol ID: 0007205/16) was approved by the Ethics Committee of Università Cattolica del Sacro Cuore, Fondazione Policlinico Universitario Agostino Gemelli (Project ID: ESR14-10185, Approval date: 24 February 2016).

#### 4.1.2. Manual DNA Isolation from FFPE Tissues

We used three different manual protocols: phenol-chloroform (PC), the GeneRead^^®^^ DNA FFPE kit (GR) (Qiagen, Hilden, Germany) and the MagMAX FFPE Total Nucleic Acid Isolation Kit (MM), (Thermo Fisher Inc.). All tissues, except for those used with the MM kit, were lysed with proteinase K digestion. 

The PC procedure was carried out as briefly described [23]. FFPE sections were scraped and digested using 33 μL 10× SDS/PK (Sodium Dodecyl Sulfate/Protein Kinase, 60 μL proteinase K and 270 μL TE (Tris EDTA-9 buffer (1 M Tris-HCl, pH 9.0; 0.5 M EDTA (Ethylenediamine tetraacetic acid), pH 8.0; 5 M NaCl; dH_2_O) and incubated at 48 °C for 12 h. After digestion, phenol/chloroform/isoamyl alcohol (25:24:1, Thermo Fisher Inc.) was added to the mixture and centrifuged for 20 min at 2500 rpm. DNA from the aqueous layer was precipitated by adding 150 μL of 7.5 M ammonium acetate (NH_4_Ac) and 900 μL of absolute ethanol. After centrifugation for 30′ at 14,000 rpm at 4 °C, the pellet was washed with 1 mL 70% cold ethanol and spun at 14,000 rpm for 10′ at 4°C. Finally, the supernatant was discarded carefully and the pellet was left to dry at room temperature and then re-suspended in 50 μL of LoTE (low-salt Tris EDTA) buffer (1 M Tris-Cl pH 7.5; 10 mM Tris-Cl pH 7.5; 0.2 M EDTA, 20 mM CaCl_2_, dH_2_O).

The GR method is based on exchange resins methodology and used the following standard protocol from Qiagen [24]. FFPE sections for each sample were scraped and re-suspended in a sterile tube with 160 μL of deparaffinization solution for 3′ at 56 °C to remove the excess paraffin. A master mix of 25 μL buffer FTB and 20 μL proteinase K was added, followed by an additional incubation at 90 °C for 60′. Finally, 35 μL of Uracil-N-Glycosilase (UNG) and 2 μL of RNAse A (100 mg/mL) were added to the mixture and incubated for 2′ at room temperature to avoid RNA contamination. In the following two steps, 250 μL AL Buffer and ethanol were consecutively added to samples and the entire lysate was transferred to the QIAamp MinElute column. The nucleic acid was adsorbed to the membrane of the QIAamp MinElute column and then washed with 500 μL AW1 and AW2 buffers, respectively. Finally, 50 μL of ATE buffer was added to the center of the membrane in order to complete the elution of DNA.

The third extraction method of MM worked using a magnetic bead-based methodology and, unlike conventional extraction methods, required no deparaffinization step. DNA isolation was performed manually and described in the following steps: sample preparation, DNA binding to magnetic beads, RNAse treatment, DNA washing and elution. To promote lysis and cell digestion, 150 μL of digestion buffer, 4 μL of proteinase K and 30 μL DNA digestion additive were added to FFPE sections and exposed to a double incubation at 60 °C for 60′ and 80 °C for 30′, respectively. Thereafter, 620 μL binding solution (200 μL binding buffer + 420 μL 100% isopropanol) was added to facilitate the binding to the precipitated DNA. In the next step, 20 μL nucleic acid binding beads was transferred for each sample and moved into a thermo-shaker for 2′. After placing the samples on the magnetic support, an electrostatic force was applied to bind the genomic DNA to the magnetic beads. Wash solutions 1 and 2 were used to clean the magnetic beads and a mixture of 99 μL nuclease-free water and 1 μL RNAse was then added to each sample for the decontamination treatment. After rapidly shaking, 100 μL 100% isopropanol was added. Finally, an elution step with 70 μL elution buffer followed by heating at 80 °C was performed to ensure the separation of the magnetic beads. 

#### 4.1.3. Automated DNA Isolation from FFPE Tissues

The two automated DNA extraction systems are known as the King Fisher Duo (Thermo Fisher Inc., Waltham, MA, USA) and the OMNIA Prima (Masmec S.p.A., Modugno (BA), 70026, Italy) stations, which work with the same solutions and magnetic bead based purification tools of the MM kit (Thermo Fisher, Waltham, MA, USA).

Robotic DNA isolation using the King Fisher Duo Instrument (Life Technologies, Thermo Fisher Scientific Inc.) was carried out in accordance with the manufacturer’s instructions. FFPE sections and 150 μL of digestion buffer were well-mixed together and transferred into a MagMax Express-96 Deep Plate. Four microliters of proteinase K and 30 μL of DNA digestion additive were added to each sample well and incubated at 60 °C for 60′ and 80 °C for 30′. After incubation, 620 μL of binding solution was added to each sample and the plate was left at room temperature for 5′ and then centrifuged. After centrifugation, 20 μL nucleic acid binding beads was transferred into each sample well. For the first step, the MagMAX plate was loaded into the instrument as follows (from row A to G); row A: 200 μL RNase solution; row B: King Fisher Duo 12-Tip Comb; into rows C, D, E: wash solution 2; row F: wash solution 1 and row G: empty. After this first DNA extraction step, a total of 600 μL of rebinding solution (400 μL isopropanol and 200 μL rebinding buffer) was placed into each DNA sample well. At the end of the run, the eluted DNA was transferred into an elution plate and stored at −20 °C.

**Figure 10:**
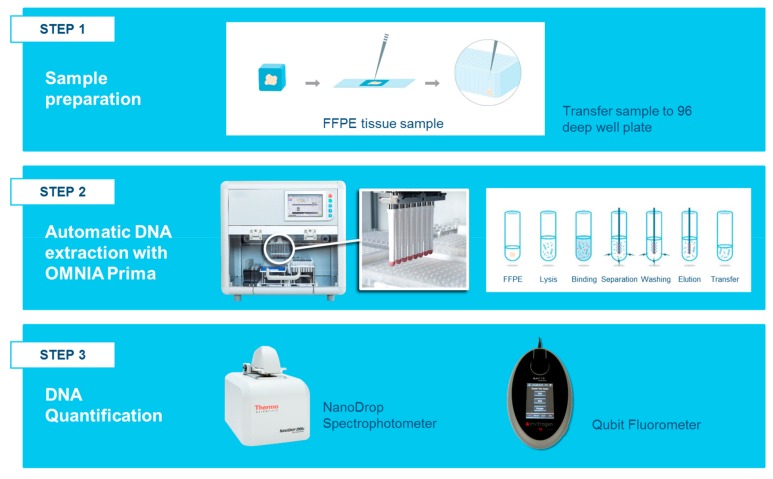
Workflow DNA analysis using the OMNIA Prima automated workstation. Step 1 shows the manual transfer to a well of the processing plate (96 deep-well plate) for each sample. Step 2 represents the fully automatic DNA extraction by the OMNIA Prima workstation. The magnetic tool of the workstation was used to catch the DNA charged beads and to wash and elute the DNA. Step 3 indicates the preliminary evaluation methods used to quantify the nucleic acids before NGS library preparation.

The OMNIA Prima automated workstation was designed and produced by MASMEC Biomed (division of MASMEC S.p.A.) and was used in association with the MM Kit. OMNIA Prima was equipped with a single pipette and a magnetic tool for analyzing 12/24 samples at the same time and six decks for 96 well plates and different size tips. FFPE sections (10 μm each) were mixed with 150 μL digestion buffer, 4 μL protease and 30 μL DNA digestion additive in a 96 deep-well plate and incubated for two steps: 60 °C for 60′ and 80 °C for 30′ in accordance with the manufacturer’s instructions. Samples were then transferred into a new 96 deep-well plate (row A) and a binding solution (200 μL binding buffer and 420 μL isopropanol) was added to each sample and incubated for 5′ at room temperature. After the incubation step, the 96 deep-well plate was loaded with different wash solutions as follows: wash solution 1 (row B) and wash solution 2 (row C). Twenty microliters of nucleic acid binding beads were transferred into each sample well and the magnetic tool of the workstation caught the DNA charged beads following washes. After RNase treatment, 400 μL isopropanol and 200 μL rebinding buffer (rebinding solution) was added to each DNA sample well and incubated on a thermo-shaker tato 1300 rpm for 5′. Samples were washed in wash solution 2 and then eluted in 75 µL of pre-heated elution buffer (Figure 10).

#### 4.1.4. Quality and Quantity DNA Assessment

DNA evaluation was performed using three different comparison methods: an Agilent 2200 TapeStation System (Agilent Technologies, Santa Clara, CA, USA), a NanoDrop/ND-1000 (Thermo Fisher) and a Qubit 3.0 Fluorometer (Thermo Fisher Inc.).

The Agilent 2200 TapeStation System is an automated reproducible method for evaluating the integrity and quantity of genomic DNA by capillary electrophoretic separation. The genomic DNA ScreenTape system analyzes genomic DNA samples in the size range from 200 bp to >60,000 bp. The ScreenTape consists of 16 samples loaded within one run and the results are available in approximately 2 h and 30′. Fresh gel-dye matrix mixture (20 μL of dye concentrate and 400 μL of gel matrix) was spin-filtered and loaded on a LabChip. Five microliters of DNA marker were added to each sample well and 1μL of DNA ladder was transferred to the assigned ladder well. Finally, 1 μL of sample was pipetted into the remaining wells. The 2200 TapeStation Analysis Software generates a value referred to as the DNA integrity number. The DIN is a decimal number ranging from 1 to 10, where 1 is attributed to completely degraded DNA and 10 to intact DNA samples. The DIN makes the interpretation of electropherograms easier, facilitates the comparison of samples and provides the repeatability of experiments and quantitation of high-quality genomic DNA for NGS technology.

NanoDrop technology is based on measuring the absorbance of small volume samples of nucleic acids and proteins. According to Beer–Lambert law, the sample retention principle employs surface tension to hold a droplet in place between two optical pedestals without the need for cuvettes or capillaries. It allows for a superior accuracy for low concentrations of DNA and ensures a much better reproducibility for higher concentrations. When assessing nucleic acid purity, the ND-1000 automatically measures a wavelength spectrum in the range from 220 nm to 350 nm. The ratio of the absorbance at 260 nm and 280 nm was used to define DNA purity which is appreciably approximately 1.8–2.

The Qubit 3.0 Flurometer ensures highly reproducibility and uses a fluorescent dye for specific dsDNA binding The Qubit 3.0 generates concentration data based on the relationship between two DNA samples of known concentration by comparing to the calibration standard. It measures total DNA concentration in terms of ng/μL of elution buffer ranging from 2 ng/μL to 1000 ng/μL. The NanoDrop overestimated DNA concentrations compared to the Qubit and its consistency with double stranded DNA (dsDNA) quantification by qPCR was restricted to a high molecular weight of DNA from FFPE samples. The Qubit DNA HS (high sensitivity) assay kit (Life Technology, Thermo Fisher Scientific) was used in accordance with the manufacturer’s instructions. Samples were prepared as follows: 2–5 µL of stock DNA or 1:5 diluted DNA was added to 195–198 µL of the Qubit working solution for a final volume of 200 µL. After incubation at room temperature for 2′, the quantification was performed by a Qubit 3.0 fluorometer.

#### 4.1.5. Assessment of PCR Amplifiable Fragment Length

The GeneRead DNA QuantiMIZE Assay (Qiagen, Hilden, Germany) is a qPCR-based approach to determine the quantity and quality of amplifiable genomic DNA from FFPE samples. This system allows us to optimize the number of qPCR cycles and DNA input for NGS target enrichment to rescue low-quality DNA. The GeneRead DNA QuantiMIZE system, according to manufacturer’s handbook, uses two qPCR assays (Assays 100 or 200, generating amplicon sizes around 100 bp and 200 bp, respectively) to study more than 40 discrete genomic loci that are randomly distributed in the genome. After DNA extraction, as long as the genomic DNA concentration was between 10 pg/µL to 2.5 µg/µL, the sample was mixed with qPCR master mix and QuantiMIZE assays were loaded into the plate. To estimate the quality and quantity of DNA, the QuantiMIZE Kit generates Ct (threshold cycle) values for each assay and provides a QC score to classify samples as having “high “or “low“ quality (≤0.04 intact DNA; >0.04 highly fragmented/damaged DNA).

#### 4.1.6. Genomic DNA extraction

Genomic DNA was extracted using an automatic station (MagCore HF16 Plus, Diatech Lab Line, Jesi, Italy). DNA concentration and quality were determined by a Nano Photometer TM (Implen, Munchen, Germany) and stored at −20 °C until use. DNA samples were processed if they met the following criteria: OD260/280 ratio = ≥1.7, concentration = ≥15 ng/μL, no degradation signals visible on agarose gel, were processed.

### 4.2. NGS Workflow

#### 4.2.1. NGS Library Preparation, Quality and Quantity Assessment

To compare the efficiency of the manual and automatic workflow of sample management to address NGS needs, DNA extracted from FFPE samples with MM manual and automatic procedures were analyzed using the AmpliSeq Colon and Lung panel v2.0 (ThermoFisher Scientific) was used. The quality check of the libraries was performed by High Sensitivity D5000 ScreenTape on the 2200 TapeStation System (Agilent Technologies). Library quantification was assessed using a Qubit^®^ 3.0 Fluorometer (Thermo Fisher, Waltham, MA, USA) using the Qubit dsDNA HS (high sensitivity) assay kit (Life Technology, Thermo Fisher Scientific).

The DNA extracted from peripheral blood (PB) for the the BRCA MASTR Dx (Multiplicom, Niel, Belgium) assay for the identification of *BRCA1* and *BRCA2* mutations was used following the manufacturers’ instructions provided by Multiplicom (Agilent Technologies, Santa Clara, CA, USA). Briefly, in the first step, all coding regions of *BRCA1* and *BRCA2* were amplified in five separate multiplex PCR amplification reactions (Plex 1–5; 93 amplicons) per individual, using a hot-start DNA polymerase. In the second step, a second round of Universal PCR was performed enabling tagging of the amplicons with specific MIDs (Molecular Identifiers) and adaptors required for sequencing. The complete protocol for *BRCA1/2* gene amplification using the BRCA MASTR Dx (Multiplicom, Niel, Belgium) assay was performed using the OMNIA Liquid Handling (LH) 100 and OMNIA LH 75 automated workstations designed and produced by MASMEC Biomed (Modugno, Bari, Italy). All steps were automated except for those involving incubation in a thermocycler (PCR plate sealing, vortexing, centrifugation). The LH 100 was equipped with a robot that can travel through the 3 spatial axes X-Y-Z, 8 independent pipette channels and a layout with two racks for reagents and DNA samples, 9 deck positions for 96 well plates and different size tips and two heating–cooling units for controlled temperature steps. This platform was used for the first step of DNA library preparation. The LH 75 was prepared with a single pipette and a magnetic tool for analyzing 12 samples at the same time and 6 deck positions for 96-well plates and different size tips. This workstation is used for DNA library pooling and purification. Both workstations were controlled by MASMEC Framework software and were provided with a UV lamp for decontamination to reduce the risk of cross-contamination.

At the same time, the manual preparation of libraries for the same samples was carried out. The quality check of PCR-enriched barcoded amplicons, for both the manual and automated libraries, was performed by PCR fluorescent labeling and fragment analysis (GS labeling QC). The GS QC assay (Multiplicom, Niel, Belgium) was carried out on an Applied Biosystem 3500 Genetic Analyzer (Life Technologies, Carlsbad, CA, USA) as previously reported by Concolino et al., 2014. MAQ-S software (Multiplicom, Agilent Technologies, Santa Clara, CA, USA) was employed for analysis results using the provided Assay Description file (.enc) and GS Reference Pattern [25].

The uniformity of manual and automated libraries was evaluated by determining the final concentration of DNA using the PicoGreen^®^ dsDNA quantitation assay (Thermo Fisher Scientific, Inc), following the manufacturers’ instructions. The assay was carried out on a LightCycler^®^ 480 Real-Time PCR System (Roche Diagnostics, Basel, Switzerland).

#### 4.2.2. NGS Analysis

Manual and automated libraries from FFPE samples were pooled in equimolar concentrations and sequenced using an Ion 540 chip on the Ion S5 GeneStudio sequencer (Thermo Fisher Scientific). After sequencing, data were automatically transferred and analyzed on the Ion Reporter Server (Thermo Fisher Scientific) using the “Colon&Lung” workflow in order to detect and annotate variants. Counts are normalized to the total number of mapped reads and expressed in reads per million.

After the GS QC assay and quantification, the libraries were sequenced using the Illumina MiSeq platform (Illumina, San Diego, CA, USA) following our molecular diagnostic routine validated setting [26,27,28]. After NGS, the sequencing fastq.gz files from MiSeq were analyzed with a CE-IVD (CE-marked In Vitro Diagnostic Medical Device) bioinformatics tool, Amplicon Suite (SmartSeq srl, Novara, Italy), to investigate the coverage parameters. Statistical data analysis of the library’s concentration and read coverage was performed using STATA software (Stata Corp. 2011. Stata Statistical Software: release 12. College Station, TX: StataCorp LP, Lakeway Drive College Station, Texas, TX, USA). Data were compared by parametric *t*-test with a cut-off of significance at *p* < 0.05.

## 5. Conclusions

Some recent papers have recently highlighted the need for standardization and harmonization of molecular pipelines surrounding NGS assays, particularly when using FFPE samples [29,30]. Automation can really improve the quality of NGS analysis by reducing pre-analytical and analytical biases due to the manual processing of molecular pipelines. In this regard, we underline how the automated workflow reported herein significantly reduced the variability of manual processing and human-related errors, providing a more reproducible process. Finally, we were able to significantly increase the throughput of our pipeline, switching from one to two runs per week.

## Figures and Tables

**Table 1:** Quantification of normal vs. tumoral DNA extracted after 24 h, 48 h or 72h of formalin fixation by three different manual recovery systems using spectrophotometric (NanoDrop) and fluorometric (Qubit) assays.

Sample	Extraction Method	NanoDrop (DNA, ng/µL)	Total DNA (ng) NanoDrop	260/280 Ratio	Qubit (DNA, ng/µL)	Total DNA (ng) Qubit
24N	MM	35.9	2513	1.83	12.4	868
24T	MM	126.4	8848	1.74	26	1820
48N	MM	64.8	4536	1.74	30.4	2128
48T	MM	103	7210	1.78	36.6	2562
72N	MM	58.9	4123	1.73	29	2030
72T	MM	73	5110	1.76	37.8	2646
24N	PC	171	3420	1.99	46.8	936
24T	PC	623.6	12472	1.94	12.92	258.4
48N	PC	156	3120	1.98	224	4480
48T	PC	325.4	6508	1.98	154	3080
72N	PC	546.6	10932	1.95	56	1120
72T	PC	106.8	2136	2.07	4.6	92
24N	GR	305.2	9156	1.95	74.4	2232
24T	GR	647.4	19422	1.91	60.4	1812
48N	GR	211.6	6348	1.93	244	7320
48T	GR	393.6	11808	1.92	117.6	3528
72N	GR	565.6	16968	1.9	112.8	3384
72T	GR	194	5820	1.97	37.6	1128

MM, MagMAX^TM^ Formalin-Fixed Paraffin-Embedded (FFPE) Total Nucleic Acid Isolation Kit (Thermo Fisher); PC, phenol-chloroform standard method; GR, GeneRead DNA FFPE Kit (Qiagen); NGS, next generation sequencing; ng, nanogram; μL, microliter; N, normal; T, tumoral.

**Table 2:** Quantification of normal vs. tumor DNA extracted after 24 h, 48 h or 72 h formalin fixation by comparing two automatic recovery systems using Nanodrop and Qubit assays.

Sample	DNA Extraction Method	DNA (ng/µL) Nanodrop	DNA (ng) Nanodrop	260/280 Ratio	DNA (ng/µL) Qubit	DNA (ng) Qubit
24N	MM-O	65.6	4920	1.6	8.94	670.5
24T	MM-O	71.2	5340	1.62	8.48	636
48N	MM-O	48.7	3648.8	1.63	4.28	321
48T	MM-O	229.2	17190	1.84	77.2	5790
72N	MM-O	297.4	22305	1.8	110	8250
72T	MM-O	75.7	56738	1.71	16.6	1245
24N	MM-KF	43.1	2155	1.7	8.34	417
24T	MM-KF	92.6	4630	1.75	30.8	1540
48N	MM-KF	51.6	2580	1.67	13.5	675
48T	MM-KF	66.7	3335	1.71	23.6	1180
72N	MM-KF	73.1	3655	1.75	24	1200
72T	MM-KF	22.8	1140	1.51	5.76	288

MM-O, MagMAX^TM^ FFPE Total Nucleic Acid Isolation Kit (Thermo Fisher), automatic procedure using OMNIA Prima (MASMEC S.p.A); MM-KF, MagMAX^TM^ FFPE Total Nucleic Acid Isolation Kit (Thermo Fisher), automatic procedure using King Fisher Duo (Thermo Fisher Inc.); ng, nanogram; µL, microliter.

**Table 3:** Quantity evaluation using spectrophotometric (NanoDrop) and fluorometric (Qubit) assays. OMNIA Prima (MM-O) vs. MM King Fisher (MM-KF).

Sample	Extraction Method	DNA (ng/µL) NanoDrop	DNA (ng) NanoDrop	260/280	DNA (ng/µL) Qubit	DNA (ng) Qubit
24N	MM-O	65.6	4920	1.6	8.94	670.5
24T	MM-O	71.2	5340	1.62	8.48	636
48N	MM-O	48.7	3648.8	1.63	4.28	321
48T	MM-O	229.2	17190	1.84	77.2	5790
72N	MM-O	297.4	22305	1.8	110	8250
72T	MM-O	75.7	5673,8	1.71	16.6	1245
24N	MM-KF	43.1	2155	1.7	8.34	417
24T	MM-KF	92.6	4630	1.75	30.8	1540
48N	MM-KF	51.6	2580	1.67	13.5	675
48T	MM-KF	66.7	3335	1.71	23.6	1180
72N	MM-KF	73.1	3655	1.75	24	1200
72T	MM-KF	22.8	1140	1.51	5.76	288

MM-O, MagMAX^TM^ FFPE Total Nucleic Acid Isolation Kit (Thermo Fisher), automatic procedure using OMNIA Prima (MASMEC S.p.A); MM-KF, MagMAX^TM^ FFPE Total Nucleic Acid Isolation Kit (Thermo Fisher), automatic procedure using King Fisher Duo (Thermo Fisher Inc.); ng, nanograms; µL, microliter.

**Table 4:** DNA quality evaluation using fluorescent capillary electrophoresis (TapeStation) and real-time PCR quantification (Quanti-Mize).

Sample	DNA Extraction Method	DIN Value	QC Score
24N	MM	5.5	−0.04
24T	MM	5.9	−0.04
48N	MM	5.4	−0.04
48T	MM	5.6	0.15
72N	MM	5.7	−0.04
72T	MM	5.9	−0.04
24N	FC	6.1	−0.03
24T	FC	nv	−0.04
48N	FC	1.9	−0.02
48T	FC	5.8	0.01
72N	FC	5.7	0.01
72T	FC	nv	−0.01
24N	GR	5.3	0.12
24T	GR	5.7	−0.03
48N	GR	4.2	−0.02
48T	GR	5.5	−0.01
72N	GR	5.7	−0.01
72T	GR	4.5	−0.01
24N	MM-O	5.6	−0.04
24T	MM-O	5.1	−0.04
48N	MM-O	4.4	−0.04
48T	MM-O	6.2	−0.03
72N	MM-O	6.2	−0.04
72T	MM-O	6.2	−0.02
24N	MM-KF	4.7	0.01
24T	MM-KF	5.8	0.00
48N	MM-KF	4.8	0.03
48T	MM-KF	4.8	0.03
72N	MM-KF	4.8	0.04
72T	MM-KF	4.1	0.03

MM-O, MagMAX^TM^ FFPE Total Nucleic Acid Isolation Kit (Thermo Fisher), automatic procedure using OMNIA Prima (MASMEC S.p.A); MM-KF, MagMAX^TM^ FFPE Total Nucleic Acid Isolation Kit (Thermo Fisher), automatic procedure using King Fisher Duo (Thermo Fisher Inc.); MM, MagMAX^TM^ FFPE Total Nucleic Acid Isolation Kit (Thermo Fisher); PC, phenol-chloroform method; GR, GeneRead DNA FFPE Kit (Qiagen); DIN, DNA integrity number; QC score, quality check score.

**Table 5:** Comparison of performance parameters of libraries prepared by manual and automated methods.

NGS Variables	Automated	Manual
Cluster passing filter	92%	85.9%
Q30 score	85	80
Error rate	0.15%	0.18%

All comparisons were not significantly different (*p* > 0.05).
